# Environmental filtering and dispersal limitation jointly shape the taxonomic, functional and phylogenetic diversity in a subtropical karst forest of China

**DOI:** 10.3389/fpls.2025.1655071

**Published:** 2025-08-28

**Authors:** Qingzhi Long, Zhili Zhan, Hu Du, Wanxia Peng, Liang Su, Hao Zhang, Zhaoxia Zeng, Fuping Zeng, Weining Tan, Youwang Mo, Xichao Deng, Yanjun Xie, Kelin Wang

**Affiliations:** ^1^ Institute of Subtropical Agriculture, Chinese Academy of Sciences, Changsha, Hunan, China; ^2^ College of Life and Environmental Sciences, Central South University of Forestry and Technology, Changsha, Hunan, China; ^3^ Huanjiang Agriculture Ecosystem Observation and Research Station of Guangxi, Guangxi Key Laboratory of Karst Ecological Processes and Services, Huanjiang Observation and Research Station for Karst Ecosystems, Chinese Academy of Sciences, Guangxi, China; ^4^ College of Resources, Hunan Agricultural University, Changsha, Hunan, China; ^5^ Management Center for Guangxi Mulun National Nature Reserve, Guangxi, China; ^6^ College of Chemistry and Biology Engineering, Hechi University, Guangxi, China

**Keywords:** multidimensional diversity, abiotic filtering, habitat heterogeneity, functional trait, karst ecosystem

## Abstract

**Introduction:**

Community assembly involves species forming communities through interactions and environmental adaptation, with traits and phylogeny playing key roles. Analyzing these factors is crucial for understanding community assembly and improving ecological restoration and biodiversity conservation, especially in karst ecosystems, where research is limited.

**Methods:**

Here, we evaluated six metrics of taxonomic, phylogenetic and functional diversity in a subtropical climax forest, and then derived the relative contribution of environmental and spatial conditions on the diversity metrics.

**Results:**

The results indicated that, except for the mean pairwise distance (MPD) index, all other indices exhibited a higher spatial distribution pattern on slopes compared to depressions. The MPD index, however, displayed a more homogeneous pattern, with no significant differences observed across terrains. Our findings suggest that topography has a stronger and more consistent influence on species, functional, and phylogenetic diversity than soil factors. Among these, phylogenetic diversity showed the most pronounced response to topographic variation (especially elevation, slope, and terrain wetness index), indicating that evolutionary lineage distribution is more sensitive to terrain changes than functional or species diversity. In addition, species diversity was most affected by dispersal limitation among the three types of diversity, suggesting that significant spatial variation in community composition is largely constrained by the dispersal ability of species. In contrast, phylogenetic diversity was most affected by environmental filtering, highlighting the strong selective effect of environmental conditions on community phylogeny. Functional diversity, on the other hand, showed a smaller degree of response to both filtering and dispersal, with dispersal limitation having a higher impact than environmental filtering.

**Discussion:**

This study reveals the spatial pattern of karst plant diversity in southwest China and its influencing factors, as well as the mechanism of community construction, providing a theoretical foundation and scientific basis for biodiversity conservation and vegetation restoration in karst areas.

## 1 Introduction

Community ecologists assess various measurements of biodiversity, including species diversity, trait-based diversity, and functional diversity. Among these, species diversity is the most straightforward indicator of plant diversity (Loreau et al., 2001). It quantitatively characterizes the composition and structure of plant communities, the progression of successional stages, the development of ecosystems, and habitat variability (Tilman et al., 1996). Studying species diversity provides scholars with valuable insights into the composition, dynamics, and evolution of plant communities (Zhang et al., 2000). Crucially, the functional traits of species—which determine the habitat a species occupies, the nature of its interspecific interactions, the intensity of competition it experiences, and its efficiency as a predator or prey—are fundamental to understanding community assembly and ecosystem functioning (Mcgill et al., 2006; Cadotte et al., 2011). Complementing this functional perspective, phylogenetic diversity offers a framework for hypothesizing the influence of historical evolutionary processes on contemporary communities (Webb et al., 2002). It allows for the analysis of community composition from an evolutionary perspective, and aids in examining the ecological processes that influence species coexistence (Cavender-Bares et al., 2009). The richness index, Shannon-Wiener index, Simpson index, and Pielou index, which measure species α-diversity, assess species distribution, dominance, and evenness within a community. These indices reflect the balance of competitive interactions or mutual survival among species, driven by the acquisition of survival resources (Tan et al., 2013).

Biodiversity patterns refer to the geographical distribution of biodiversity, which provides an intuitive representation of how biological and environmental factors influence species distribution (Gaston, 2000). Understanding biodiversity patterns and their driving mechanisms is essential for biodiversity conservation research (Naeem and Wright, 2003; Mcgill et al., 2006). Community assembly mechanisms are generally classified into two main categories: ecological niche processes, which focus on differences in ecological strategies among species, and neutral processes, which emphasize the similar fitness levels of species (Niu et al., 2009). Ecological niche processes encompass competition and facilitation, while neutral processes are considered stochastic. Recent studies have shown that both facilitation and competition can simultaneously influence species coexistence within the same community, particularly under the influence of abiotic environmental stresses (e.g., low temperature, nutrient deficiencies in soil, drought, etc.) (Pichon et al., 2024). The relative strength of these effects varies depending on the intensity of the environmental stresses (Kraft et al., 2008; Mcintire and Fajardo, 2009; Long et al., 2015). Abiotic environments act as fundamental environmental sieves, shaping plant morphological structures, physiological functions, and phylogenetic relationships. This filtering process fundamentally determines the pool of species capable of coexisting within a community by defining the abiotic dimensions of their ecological niches. Furthermore, by providing varying quantities and qualities of resources, abiotic environments play a crucial role in facilitating the actual coexistence among these filtered species (He et al., 2009).

Environmental filtering, along with dispersal limitation, is recognized as a critical process in community assembly (Hillerislambers et al., 2012). Environmental filtering refers to the process by which abiotic factors select species with specific traits for incorporation into local communities, often described as the “environmental sieve” (Keddy, 1992). The central concept of habitat filtering is that species exhibit varying levels of suitability across different environments, leading to differences in population sizes. It is generally accepted that when environmental filtering plays a prominent role in local communities, the mean values of functional traits and the trade-offs between traits will vary along abiotic environmental gradients. For instance, Liu et al. (2017) demonstrated that soil fertility was significantly correlated with leaf area, leaf area index, and wood density. In addition to environmental filtering, dispersal limitation at the local scale is another key process influencing forest biodiversity and community assembly (Shen et al., 2009; Chen et al., 2019). Dispersal limitation in plant communities refers to the insufficient number or variety of propagules that prevent seeds from reaching suitable germination sites (Dent and Estrada-Villegas, 2021). The significant role of dispersal limitation has been widely confirmed in previous studies, which span various forest types (temperate, subtropical, tropical), biodiversity dimensions (species diversity, phylogenetic diversity, functional diversity), and taxonomic groups (Legendre et al., 2009; Wang et al., 2018). For example, a study in the subtropical forest of Gutian Mountain found that both dispersal limitation and habitat filtering had comparable explanatory power for patterns in species and phylogenetic diversity in plant communities. Notably, the effect of dispersal limitation was more pronounced at smaller spatial scales, while the influence of habitat filtering increased with scale (Rao et al., 2013). Recent studies have explored the interplay between environmental filtering and dispersal limitation in shaping biodiversity, for example, Wang et al. (2013) and Yin et al. (2021) demonstrated the importance of these processes in temperate and tropical forests. While these studies have provided valuable insights, our research aims to explore their combined effects in the unique karst ecosystems of southwest China, where steep topography and nutrient-poor soils create distinctive community assembly patterns.

Ecological heterogeneity is a key characteristic of landscape patterns and a significant factor influencing biodiversity (Stein et al., 2014; Tamme et al., 2010). Ecological niche partitioning driven by habitat heterogeneity is particularly important in the context of abiotic factors such as light availability, nutrient resources, soil moisture content, and topographic conditions. These factors also influence the spatial and temporal distribution of vegetation (Keppel et al., 2012; Bátori et al., 2019). Studies have demonstrated that even relatively minor changes in environmental variables can have substantial effects on the species composition and diversity of plant communities (Deák et al., 2021). Topography, as a major non-biotic factor, plays a pivotal role in influencing vegetation cover through variations in slope, elevation, and aspect. These topographic features, in turn, affect the spatial redistribution of sunlight, soil moisture, and nutrients, resulting in local environmental modifications and the creation of microclimates (Cantón et al., 2004; Hara et al., 1996; Shen et al., 2000). Topographic factors exert a strong influence on community, ecosystem, and landscape patterns (Kamrani et al., 2011; Chen et al., 2018), thereby impacting biodiversity (Gaston, 2000). Beyond topography, soil nutrient availability is a critical determinant of plant growth and development, influencing vegetation distribution. For instance, soil nutrient levels are closely linked to spatial heterogeneity in grasslands (Qi et al., 2010) and natural forests (Huang et al., 2015; Toriyama et al., 2015). Moreover, research has shown that a broad range of soil nutrients, including organic matter, nitrogen, and phosphorus, significantly affects the species composition and diversity of plant communities (Townsend et al., 2008; Becknell and Powers, 2014).

Karst regions, characterized by distinct geomorphological features, are home to rich ecological diversity and complex geographical conditions (Huang, 2025). Plant communities in these areas exhibit remarkable adaptability, enabling them to thrive in nutrient-poor soils, extreme hydrological conditions, and specialized karst landscapes (Wu and Wu, 2023). Many plants in karst regions possess traits such as drought resistance and tolerance to poor soils (Huang, 2025). These species often adapt by developing deep root systems or thickened leaves to cope with local climatic and soil conditions. Due to the thin and uneven soil layers created by karst processes and the exposure of rock surfaces, plants must acclimate to nutrient-deficient environments (Liu et al., 2021). The karst landscape is characterized by complex topographical features such as karst peaks, pillars, caves, and stone forests. These formations provide diverse habitats for plants, create varied hydrological conditions, and influence the distribution of plant communities (Li et al., 2020). Regarding community assembly, research on karst plant communities has highlighted the complex dynamics shaped by species interactions—such as competition and mutualism—occurring within the context of, and often driven by, species’ adaptations to unique environmental factors (e.g., drought, high calcium, soil limitations) (Fu et al., 2023). Studies have shown that the plant communities in karst areas are closely linked to soil types, climatic conditions, and hydrological characteristics. Changes in these environmental factors directly affect species distribution and community structure (Liu et al., 2021). Furthermore, functional diversity plays a crucial role in karst plant communities, as species with varying functional traits coexist, contributing to the stability and ecological functions of the community (Wang et al., 2022). Despite extensive research on the basic characteristics and ecological processes of karst plant communities, several gaps remain in our understanding. Notably, the dynamic processes of community assembly and succession are still not fully understood (Fu et al., 2023).

In this study, we focus on subtropical karst evergreen-deciduous broadleaf mixed forests, aiming to systematically investigate and analyze species diversity, functional trait diversity, and phylogenetic diversity. Our goal is to explore the patterns of these diversities and their relationships with environmental factors. We hypothesize the following: First, species diversity, functional trait diversity, and phylogenetic diversity will exhibit distinct spatial patterns. Second, in karst regions, topography may exert a stronger influence on the distribution and diversity of plant communities compared to soil chemical properties, playing a more significant role in ecological regulation. Third, we propose that environmental filtering may play a more dominant role than dispersal limitation in the community assembly of karst plant species.

## 2 Materials and methods

### 2.1 Study site

The Mulun National Nature Reserve is located in the northwestern part of Guangxi Huanjiang Maonan Autonomous County (25°7’–25°12’N, 107°54’–108°5’E). The reserve is bordered to the south by the Yunnan-Guizhou Plateau and to the north by the Maonan National Nature Reserve in Guizhou, encompassing a total area of 10,800 hectares. The elevation within the reserve ranges from 400 to 1,000 meters. Situated within a karst landscape of peaks and depressions, the reserve is characterized by unique landforms, diverse topography, and a complex ecological environment, with evergreen-deciduous broadleaf mixed forests. The soil in this area is primarily dark or brown limestone soil, derived from carbonate rocks, exhibiting non-zonal characteristics. The soil layer is shallow, with exposed rocks, and the pH value ranges between 7.06 and 7.68. The climate is warm and humid, typical of a mid-subtropical monsoon climate, with an average annual temperature of 19.4°C. Annual rainfall ranges from 1,530 to 1,820 mm, with the majority of precipitation occurring between April and September. The region enjoys an average of 4,422 hours of sunshine annually and has a frost-free period of 290 days (Lu et al., 2021a).

A fixed permanent monitoring plot of 25 hm² (500 m × 500 m) was established in the reserve in 2014, following the CTFS (Centre for Tropical Forest Science) standards. And the plot was first re-inspected in 2019 and will be fully re-inspected every five years thereafter. The plot is situated at an elevation ranging from 442.6 to 651.4 meters. The habitat across the entire sample site is highly heterogeneous, with the typical “peaks and depressions” landscape, including mountain tops, slopes, and depressions. Dominant species within the plot include *Cryptocarya macrocarpa*, *Itoa orientalis*, *Platycarya longipes*, and *Lindera communis* (Du et al., 2017).

Prior to the investigation, the entire plot was divided into 625 sample squares (20 m × 20 m) using a total station and RTK (Real-time Kinematic, Trimble R10, USA). The diameter at breast height (DBH) and coordinates of all woody plants with a DBH ≥ 1 cm were measured, and species names, along with other relevant information, were recorded. The spatial distribution of all individuals spanned various habitat types within the plot, providing a comprehensive representation of the forest stand’s diameter structure.

### 2.2 Functional traits

A total of 144 species, each with 25 or more individuals in the plot, were selected for investigation. To ensure representative sampling and avoid overrepresentation of any single species in the dataset, 10 to 15 mature and well-developed individuals of each tree species were randomly chosen from the sample site. The spatial distribution of the selected individuals spanned various habitat types within the plot, offering a comprehensive representation of the forest stand’s diameter structure.

Following the guidelines from Pérez-Harguindeguy’s functional trait collection manual (Pérez-Harguindeguy et al., 2016) and the standards of the Center for Tropical Forest Science (CTFS), leaf samples were collected from the south, north, east, and west sides of the canopy, where there was minimal shading. A minimum of 20 leaves were collected from each selected plant. Leaf area (LA, mm²) and leaf length-width ratio (LW) were measured using the ZhongJing SM I800 Plus scanner and the Wanshen LA-S series plant image analysis system. Leaf thickness (LT, mm) was measured with a thickness gauge, ensuring that the veins were avoided during measurement. The collected leaves were placed in envelopes and dried in a laboratory oven at 60 °C until they reached a constant weight, which typically took approximately 48 hours. The dry weight of the leaves was recorded. For further analysis, the dried leaf samples were ground to a 100-mesh powder using a ball mill. Element content of the samples was determined using various methods: leaf carbon (LC) and nitrogen (LN) concentrations were analyzed with an elemental analyzer; phosphorus content was measured using ammonium molybdate spectrophotometry; and the concentrations of elements such as potassium (LK), calcium (LCa), magnesium (LMg), aluminum (LAl), iron (LFe), zinc (LZn), manganese (LMn), sodium (LNa), and sulfur (LS) were analyzed using an ICP-OES 5110 instrument.

### 2.3 Phylogenetic tree

The plastid genomes of these species were sequenced from silica gel-dried materials collected from one to two individuals of each tree species at the sampling site (Pahlich and Gerlitz, 1980). In total, 209 new plastomes were generated, representing 209 species, 147 genera, and 61 families. Based on the coding regions of these 209 plastomes, a megaphylogeny was constructed using the maximum likelihood (ML) approach in RAxML v8.2.12 (Stamatakis, 2006). The phylogeny was then dated using the penalized likelihood method in treePL (Smith and O’meara, 2012). The raw sequencing data for all the plastid genomes generated in this study have been submitted to the NCBI Sequence Read Archive (SRA) under the accession numbers SRX22362678 to SRX22362939 (Jin et al., 2023).

### 2.4 Environmental variables

To establish a grid system, Real-time Kinematic (RTK) and total station measurements were used to mark and fix 10 m × 10 m grid points, resulting in a total of 2,601 points. The average elevation (ELE) of each quadrat (20 m × 20 m) was calculated by averaging the elevations of the four corner points. The slope (SLO) and slope aspect (ASP) within each quadrat were measured using a compass. Concavity (CON) was determined as the difference in elevation between the average elevation of the target quadrat and the average elevation of the surrounding eight quadrats. The rock outcrop rate (ROC) was quantified in each quadrat using a standardized point-intercept sampling method. At systematically distributed grid points (n = 100 points per 10×10 m quadrat), substrate was classified as either bedrock outcrop or soil-covered. The terrain wetness index (TWI) was calculated using the System for Automated Geoscientific Analyses (SAGA GIS) (Lu et al., 2021b; Du et al., 2017).

For soil analysis, a total of 625 soil samples were collected from the center of the 20 m sample plots and the center of four smaller plots (five points in total). The samples were taken from the 0–10 cm surface soil layer. The collected soil samples were screened using a 2 mm mesh, air-dried in a ventilation chamber, and then ground in a ball mill for chemical analysis. Following the standard methods outlined by Long et al. (2023), several measurements of soil physicochemical properties were conducted, including soil pH, soil organic carbon (SOC), total nitrogen (TN), total phosphorus (TP), total potassium (TK), available nitrogen (AN), available phosphorus (AP), available potassium (AK), calcium, and magnesium (Su et al., 2023a).

### 2.5 Statistical analysis

Pearson correlation analysis (PCA) was conducted to assess the relationship between environmental factors and biodiversity indices. Variables were selected based on previous studies and ecological relevance (Yao et al., 2017; Qin et al., 2025). Redundancy analysis (RDA) was performed to explore the relationship between biodiversity metrics and environmental variables. Selection of variables for inclusion in the RDA was based on their significance in influencing community structure, as determined by preliminary correlation analysis. The Moran’s Eigenvector Maps (MEM) and Principal Coordinates of Neighbour Matrices (PCNM) were used to account for spatial structure in the data. Preprocessing steps included standardization of environmental variables and distance matrices for spatial autocorrelation before applying these analyses. Distance-based Moran’s eigenvector maps (dbMEM) were used as spatial variables. Variance Partitioning Analysis (VPA) was employed to partition the environmental sources of variance driving the observed differences in biodiversity indices, allowing for the testing of the proportion of variation attributable to environmental effects, interaction effects, and unexplained effects.

All analyses were conducted in R 4.2.2. The taxonomic diversity index, including Shannon Wiener Diversity Index (Shannon) and Pielou’s Evenness Index (Pielou), was calculated using the “vegan” package. The phylogenetic diversity index, including Phylogenetic Diversity (PD) and Mean Pairwise Distance (MPD), was determined using the “picante” package. And the functional diversity index, including Rao’s quadratic entropy index (RaoQ) and Functional Divergence index (FDiv), was computed using the “FD” package (Laliberté et al., 2014). The dbMEM were calculated using the “adespatial” package (Dray et al., 2020). Redundancy analysis (RDA) and variance partitioning analysis (VPA) were performed using the “vegan” package (Oksanen et al., 2015), while hierarchical partitioning analysis was conducted using the “rdacca.hp” package (Lai et al., 2022).

## 3 Results

### 3.1 Spatial patterns of α diversity

**Figure 1:**
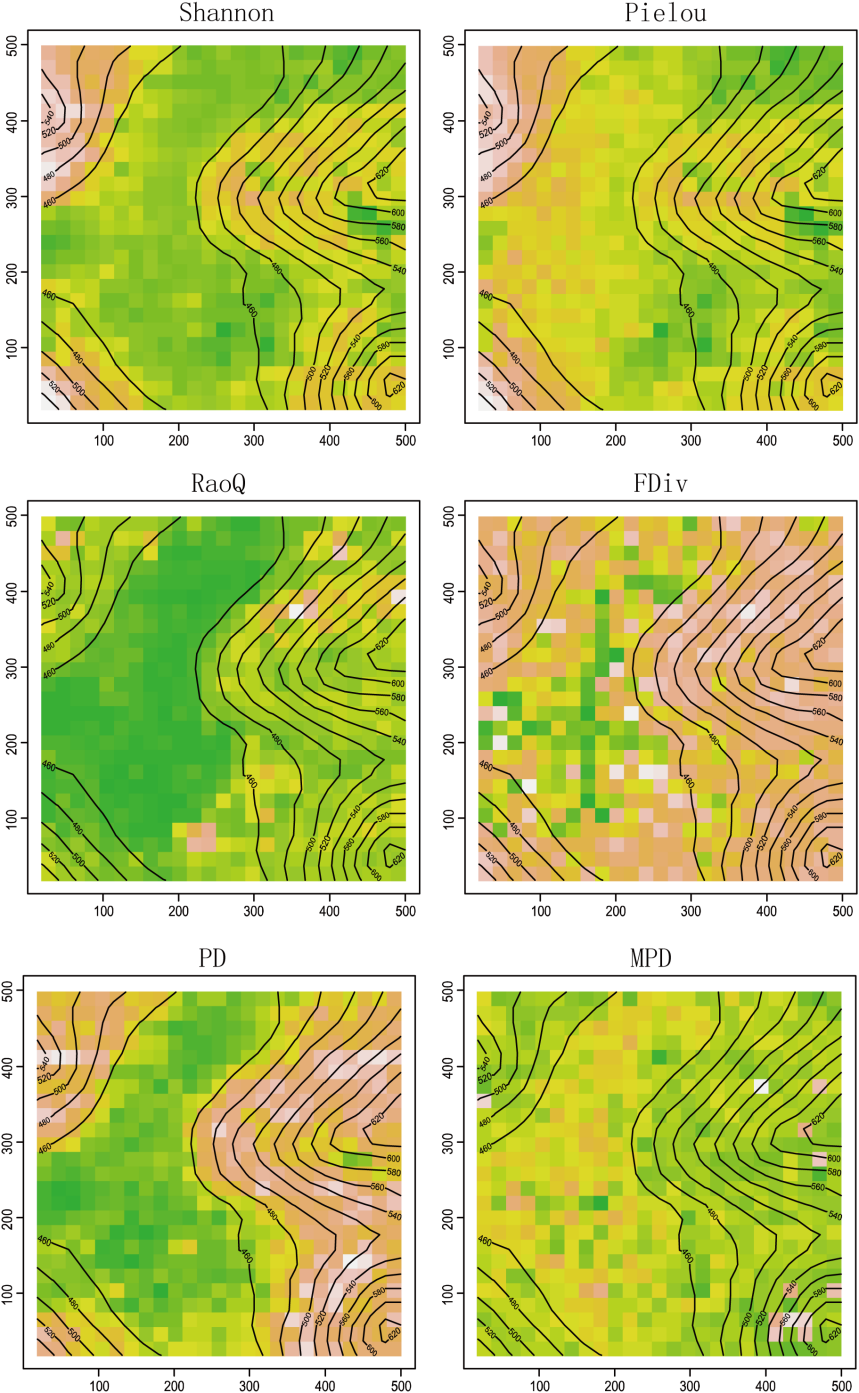
Spatial distribution of each diversity index. Green represents lowest content, followed by yellow, orange, pink, and the highest is white.

The spatial distribution of each biodiversity index (**Figure 1**) reveals that the values of PD, RaoQ, FDiv, Shannon, and Pielou are higher on the slope and lower in the depression. In contrast, MPD exhibited a more even distribution across the plot, with less variation between different habitat types. In terms of taxonomic diversity, the Pielou index was higher in the depression area. Regarding functional diversity, the difference in the FDiv index between the slope and depression was greater than that of the RaoQ index. For phylogenetic diversity, the PD index was lower in the depression area, with a greater difference between the depression and slope, whereas the MPD index showed the opposite pattern.

### 3.2 The correlation between diversity and environmental factors

**Figure 2:**
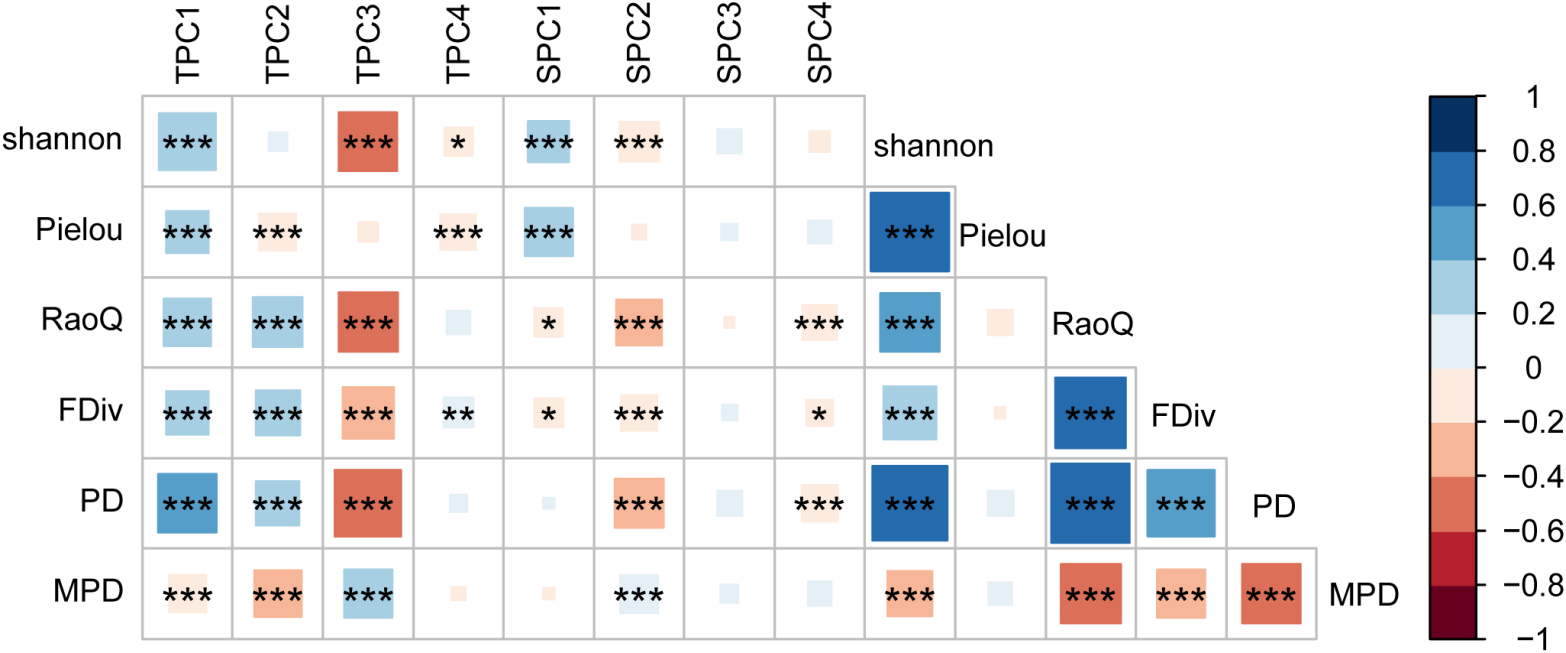
Correlation coefficients between environment factors and biodiversity. Abbreviations: TPC1, 2, 3, 4, the four axis of topographic principal component; SPC1, 2, 3, 4, the four axis of soil principal component. * P<0.05; **P<0.01; ***P<0.001.

**Figure 3:**
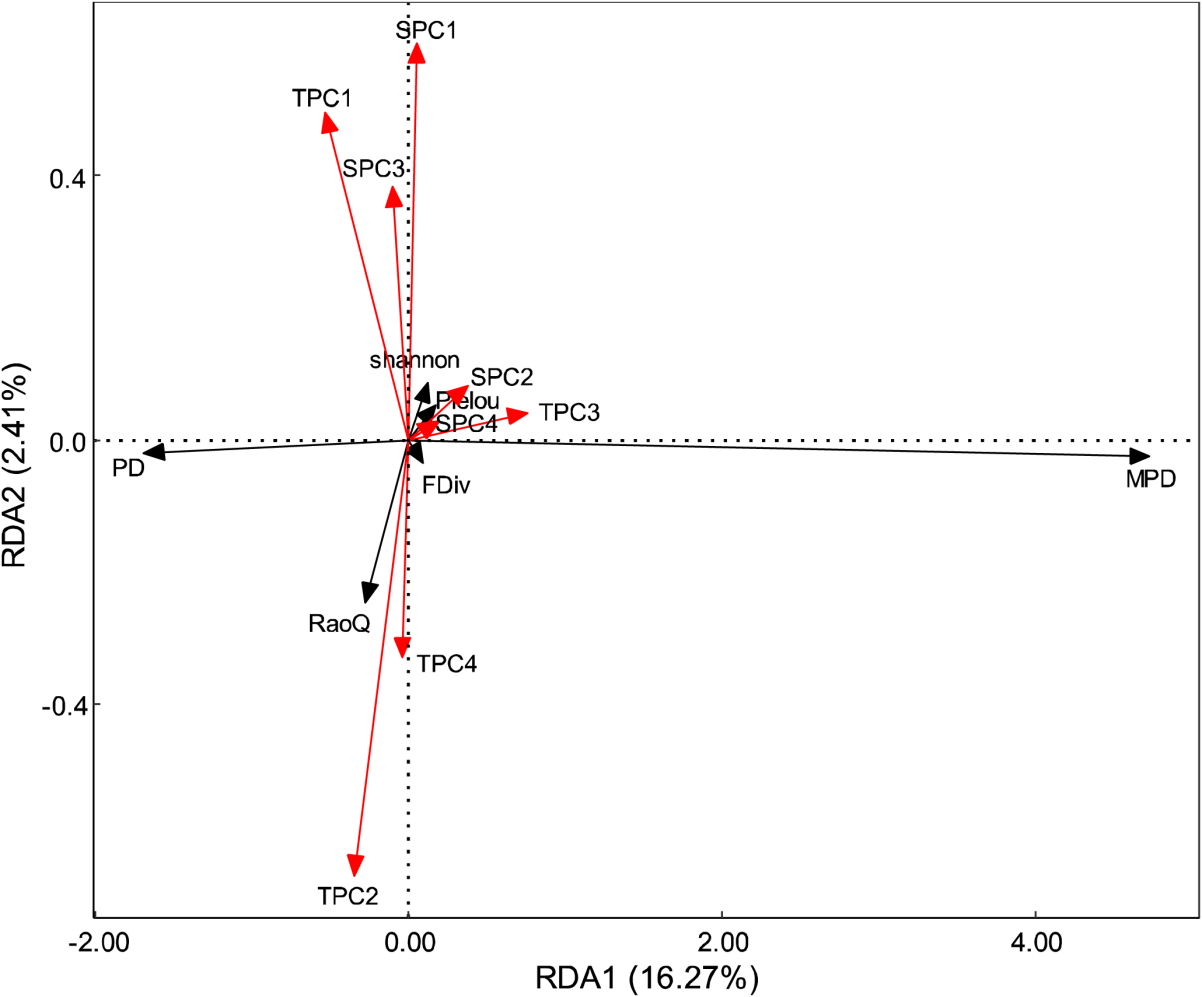
RDA analysis on the relationship between environment factors and diversity. Red arrows represent environmental factors, black arrows represent diversity.

**Figure 4:**
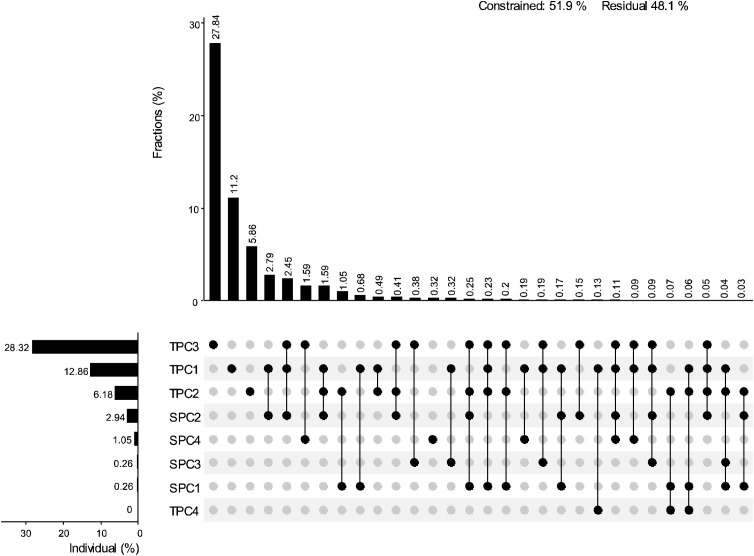
The contribution of each environmental factor to the RDA results. UpSetView plots of variation-partitioning results to show the pure and shared contributions of environment variables on diversity. The numbers in the graphs are the percentage of variance explained by the corresponding environmental factors. The dot matrix and the corresponding bar above it show the values of shared and exclusive contributions. Residuals represent the percentage unexplained by these variables. The corresponding bar on the left indicates the values contributed by each impact factor individually.

The results of the Pearson correlation analysis indicated that the correlation between topographic factors and biodiversity was generally higher than that of soil factors (**Figure 2**). The correlation pattern for the terrain axis 3 was opposite to that of the other topographic factors. Additionally, the correlation of soil axis 3 with all biodiversity indices was not significant. The correlation of MPD with environmental factors followed a trend opposite to that of the other biodiversity indices. All biodiversity indices were significantly correlated with one another, except for the Pielou index. Redundancy analysis (RDA) revealed that the first and second axes explained 16.27% and 2.41% of the variation in the biodiversity indices, respectively, with a cumulative explanation of 18.68% (**Figure 3**). The first axis accounted for most of the total variance, showing a positive correlation with MPD and a negative correlation with PD. In contrast, the RaoQ index exhibited an opposite trend to the Shannon index and contributed more to the second axis. Among all environmental factors, the topographic PC3 axis had the highest individual contribution (28.32%), while the topographic PC4 axis had the lowest individual contribution (**Figure 4**).

### 3.3 Impact of environmental and spatial factors on diversity

**Figure 5:**
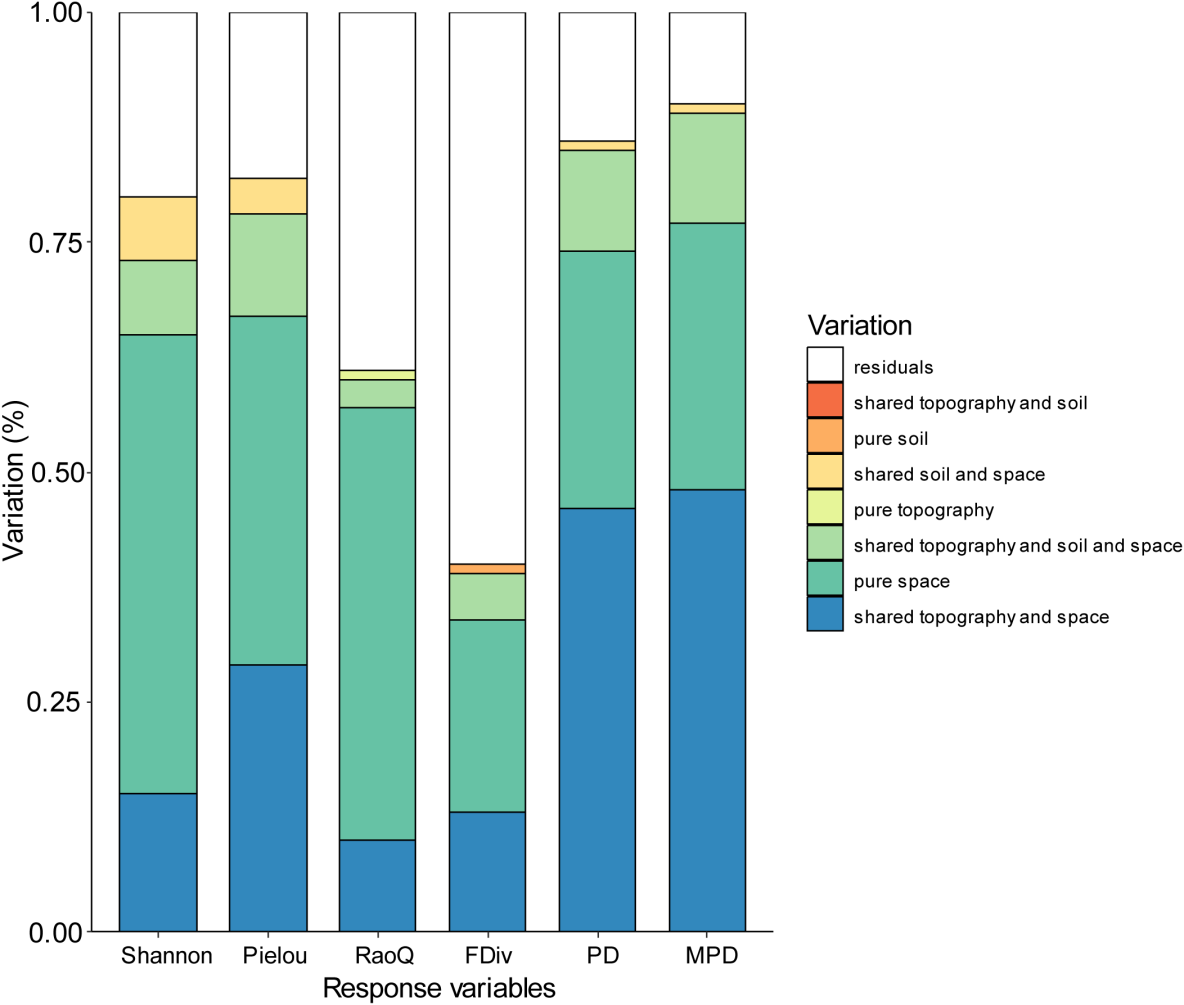
Variation partitioning results for diversity against the topographic, soil and spatial variable.

The results of the variance partitioning (VP) analysis for each biodiversity index revealed that pure space contributed most to Shannon and Pielou indices, while for PD, MPD, and RaoQ, shared topography and space contributed the most (**Figure 5**). The combined effect of the interaction between topographic and spatial factors, along with pure spatial factors, accounted for the majority of the observed variations in biodiversity indices, except for FDiv. In contrast, residuals explained most of the variation in FDiv. The contribution of soil factors to the biodiversity indices was generally low, and even the interaction between topographic and soil factors did not explain any of the observed variation across all indices.

## 4 Discussion

The pattern of species diversity in montane plant communities is primarily shaped by factors such as habitat heterogeneity, vegetation type, and topography and geomorphology (Hjort et al., 2015; Bailey et al., 2018). The distribution patterns of the Shannon and Pielou indices in this study indicate a more even distribution of species from depressions to slopes, coupled with an increase in community diversity (**Figure 1**). This pattern is attributed to the complexity of karst mountain topography, variations in slope position, and differences in the distribution of other environmental resource factors (Stein et al., 2014; Hamm and Drossel, 2017). For instance, in the low-elevation depressions and the foothills of their edges, although the soil layer is deeper and humidity conditions are more favorable, the duration of direct sunlight is limited. These areas are generally dominated by shade-loving and shade-tolerant species, leading to a less broadly adapted environment. As a result, species diversity and composition in these regions are less homogeneous than in adjacent higher-elevation slopes, where light levels are more abundant. On these higher slopes, diversity and species composition are greater (Chiu et al., 2020). The study suggests that karst depression areas, with more favorable water-heat ratios than higher-elevation slopes, are more likely to support communities with higher species richness, more complementary ecological niches, and a more even distribution across the community (Liang et al., 2004).

Compared to species diversity, functional diversity provides a more direct explanation of the roles that plants play in ecosystems. It has also been extensively tested in various ecological contexts, such as changes along latitudinal gradients, mechanisms of interspecific competition within communities, explanations of productivity, transitions across different successional stages, and global-scale modeling predictions (Díaz et al., 2007). In this study, the FDiv and RaoQ indices were higher on the slopes than in the depressions, indicating that fewer ecological niche resources are available to species in depressions compared to slopes. This results in lower functional trait differences and more intense competition for resources among species in depression areas (Legras et al., 2020).

In the plot, the PD values were higher on the slope than in the depression, with the hilltop primarily consisting of evergreen-deciduous broadleaf (including coniferous) mixed forests. The slope, serving as a transitional zone between the hilltop and depression, was characterized by the interspersed coexistence of multiple communities, which increased species richness (Su et al., 2023b). In this study, the MPD index displayed a uniform distribution pattern and was less influenced by habitat type, indicating that the Mulun Karst area has experienced minimal disturbance and remains relatively primitive in its historical development. This suggests that community evolution across different habitats in the area has been more synchronized (Zhang et al., 2010). Similar spatial patterns were observed between plant species diversity indices, functional diversity indices, and phylogenetic diversity indices, suggesting that plant communities in karst areas result from a combination of ecological niche differentiation, species interactions (e.g., competition, symbiosis), and environmental heterogeneity. Given that these diversity patterns are closely linked to factors such as elevation gradients and slope, it can be inferred that environmental factors, particularly those influenced by elevation, play a more significant role in shaping species composition, functional redundancy, and the development and maintenance of ecosystem functions in these communities (Manish, 2021).

RDA analysis revealed that all four terrain factors and three soil principal component axes significantly influenced biodiversity indices. Correlation analysis further showed that the correlation between terrain factors and biodiversity indices was stronger than that of soil factors, suggesting that terrain factors are the primary drivers of community assembly. This finding aligns with tropical forest studies, where local-scale terrain factors were found to explain more of the species diversity distribution than soil and biological factors. In terms of topography, the main contributors to the first and second axes were elevation (ELE), slope (SLO), and topographic relief (STK) (**Figure 3**; **Supplementary Table S1**), all of which were significantly positively correlated with most biodiversity indices. Elevation, as a composite of environmental factors, integrates changes in temperature, rainfall, light, and soil gradients, all of which influence community species composition and phylogenetic structure. It has been shown that tree and shrub species richness decreases with elevation, likely due to environmental changes such as lower temperatures (Cirimwami et al., 2019). Furthermore, it has been suggested that as elevation increases, plant dispersal modes shift, with wind- and insect-borne dispersal becoming more prevalent at higher elevations, whereas lower elevations primarily involve a single dispersal mode. This shift contributes to the higher impact of elevation on biodiversity (Cornwell and Ackerly, 2009). Additionally, plants at higher altitudes tend to have larger fruits, and the slope plays a role in their dispersal. Soil is fundamental to plant growth, and the relationship between soil thickness and species richness is closely linked (Jones et al., 2008). For example, Dornbush found that increased soil thickness in tallgrass prairie promotes higher species richness (Dornbush and Wilsey, 2010). Thicker soils typically support more plant species, as they retain more water and nutrients, providing a more favorable environment for growth. The main contributor to the third axis was the terrain wetness index (TWI), indicating that plants adapt to water stress by developing diverse functional traits.

For soil, the main contributors to the first axis were soil organic carbon (SOC), soil nitrogen, and calcium concentrations (**Figure 3**; **Supplementary Table S2**), suggesting that species composition within the community interacts with soil fertility. Different trait strategies among species in varying soil environments arise from plant trade-offs between growth and nutrient storage (Guilbeault-Mayers et al., 2024; Delpiano et al., 2020). Environmental change affects whether species undergo an environmental filtering process within the community, where species with specific traits, suited to each environmental filter, co-construct the community through environmental conditions and biological interactions (Viana and Dalling, 2022). The spatial heterogeneity of calcium (Ca) and other nutrient elements in soils is particularly pronounced in karst regions (Lian et al., 2023; Du et al., 2014). Studies have shown that the spatial distribution of calcium and other elements in soil is closely linked to factors such as topography, elevation, and slope (Lian et al., 2023). For example, in the study area, depressional zones at the base of slopes tended to have higher soil calcium levels, while hilltops exhibited lower levels. The spatial heterogeneity of soil nutrients influences the adaptability of different plant communities (Wang et al., 2017). Nutrient-rich areas, such as downslopes and depressions, provide more favorable environmental conditions for rapid growth and resource accumulation, thereby promoting species diversity. In contrast, hilltop areas with nutrient-poor soils favor species that can tolerate poor conditions and possess special adaptations. Overall, the spatial heterogeneity of nutrient elements and calcium in soils plays a crucial role in influencing plant diversity. It not only determines the growing conditions for plants but also shapes the structure and diversity of plant communities in karst regions by influencing the growth strategies, competitive abilities, and ecological adaptations of species.

In this study, we found that the environmental factors most strongly correlated with the biodiversity of woody plants in the region were elevation (ELE), slope (SLO), and the terrain wetness index (TWI) (**Figure 4**; **Supplementary Table S1**). These correlations may be attributed to the unique geomorphological features of the karst region, which result in significant differences in water and thermal conditions across various habitats. Water and thermal conditions have a profound influence on plant distribution, and the environmental filtering effect of large variations in these conditions significantly impacts species community structure. Additionally, we investigated the effects of individual environmental factors on plant community diversity, yielding similar conclusions to those of previous studies (**Supplementary Figure S1**). ELE was positively correlated with the Shannon and Pielou indices, suggesting that the soil and climatic conditions in higher elevation regions may promote species diversity. Similarly, changes in SLO significantly affected both the Shannon and Pielou indices, highlighting the key role of slope in influencing species diversity. Steep and gently sloped areas had differing effects on diversity, suggesting that slope variations lead to differences in growing conditions for plants, thereby affecting community structure. The limited effect of aspect (ASP), particularly on the Shannon and Pielou indices, suggests that while ASP may influence moisture and light distribution, its impact was relatively weak in this study. The TWI had a significant impact on both the Shannon and FDiv indices, indicating that moisture plays a crucial role in shaping species diversity in karst areas. Soil organic carbon (SOC) and pH also had strong effects on the Shannon and Pielou indices, underscoring the importance of soil chemical properties in determining plant community diversity, with SOC being particularly linked to species growth and distribution. Nutrient elements such as nitrogen, phosphorus, and potassium had less significant effects on the diversity indices, likely because variations in their concentrations exert less influence on plant community structure or are moderated by other environmental factors.

Several studies have found that environmental gradients strongly influence species composition in subtropical and karst regions (Chen et al., 2024; Du et al., 2017; Hu et al., 2023). While similar results were observed in the present study, the variance decomposition analysis revealed that some spatial processes contributed more to biodiversity than environmental processes. Thus, the influence of spatial processes should not be overlooked (**Figure 5**). Both the species diversity index and the functional diversity index were most strongly explained by purely spatial factors. In the karst region, spatial factors such as topography, elevation, and slope had a greater explanatory power for species diversity and functional diversity indices, indicating that the spatial distribution of species was significantly influenced by these factors. The high topographic heterogeneity and habitat complexity in karst regions resulted in a marked effect of spatial distance on species distribution. Previous studies have shown that the natural spatial gradient of habitats formed by the unique peak-and-depression landscape in karst regions strongly influences species compositional variability (Hu et al., 2024). This spatial heterogeneity may lead to habitat isolation and dispersal limitation, thereby affecting species distribution and diversity. In contrast, the phylogenetic diversity index was more strongly explained by environmental factors. Phylogenetic diversity is closely related to the evolutionary history and ecological adaptations of species, and environmental conditions (e.g., soil type, moisture, and temperature) play a dominant role in shaping phylogenetic diversity by influencing species’ ecological adaptations and population distributions (Lv et al., 2024). Changes in environmental conditions affect the phylogenetic diversity of species by determining the growth patterns of certain species and their ability to adapt to different habitats.

Functional traits, as objective phenotypes of plants, can directly quantify ecological adaptations. In this study, it was found that spatial factors had a greater explanatory power for the RaoQ index, confirming that, due to the complexity of habitats in karst regions, the functional traits of different species are influenced by spatial effect gradients, leading to varying forms of adaptation (Zhang et al., 2022; Long et al., 2023). Phylogenetic analysis, based on species affinities, can estimate the ecological similarity among species. In this study, the combined effects of topography and spatial factors had the most significant impact on phylogenetic diversity. This is due to the habitat gradient created by the crested depressions in the karst region, which has pronounced spatial effects. Overall, biodiversity in karst areas is strongly influenced by both spatial and mixed factors. This finding is consistent with most studies, as larger spatial distances often correspond to greater environmental distances. The effect of dispersal limitation, primarily manifested through the purely spatial component, also exerts a non-negligible influence on biodiversity.

## 5 Conclusions

This study integrates taxonomy, functional traits, and phylogeny to examine the spatial distribution of biodiversity in karst regions. The observed differences in the distribution of various diversity measures reflect plant adaptations to specific karst habitats and the outcomes of long-term evolutionary processes. We found that different types of diversity are driven by distinct environmental factors, which lead to variations in the distribution of diversity indices. Additionally, we analyzed the relative contributions of environmental and spatial processes in subtropical karst forests. The results indicate that terrain factors have a stronger correlation with biodiversity than soil factors. Overall, elevation, slope, and the topographic wetness index showed higher correlations with biodiversity. Both environmental and spatial factors jointly contribute to the spatial patterns of biodiversity. Dispersal limitation has a greater impact on species and functional diversity, while environmental filtering predominantly influences phylogenetic diversity. Based on these findings, we propose that environmental filtering and dispersal limitation together govern community assembly in woody plants in karst regions. Our results highlight the relationship between ecological niches and spatial processes in shaping species diversity, emphasizing the critical role of terrain factors in determining the community structure of karst forests. These findings provide a scientific foundation for understanding community assembly processes in karst regions.

## Data Availability

The original contributions presented in the study are included in the article/**Supplementary Material**. Further inquiries can be directed to the corresponding author.

## References

[B1] Bailey J. J. Boyd D. S. Field R. (2018). Models of upland species’ distributions are improved by accounting for geodiversity. Landscape Ecol. 33, 2071–2087. doi: 10.1007/s10980-018-0723-z, PMID: 30930538 PMC6404796

[B2] Bátori Z. Vojtkó A. Maák I. E. Lőrinczi G. Farkas T. Kántor N.. (2019). Karst dolines provide diverse microhabitats for different functional groups in multiple phyla. Sci. Rep. 9, 7176. doi: 10.1038/s41598-019-43603-x, PMID: 31073136 PMC6509348

[B3] Becknell J. M. Powers J. S. (2014). Stand age and soils as drivers of plant functional traits and aboveground biomass in secondary tropical dry forest. Can. J. For. Res. 44, 604–613. doi: 10.1139/cjfr-2013-0331

[B4] Cadotte M. W. Carscadden K. Mirotchnick N. (2011). Beyond species: Functional diversity and the maintenance of ecological processes and services. J. Appl. Ecol. 48, 1079–1087. doi: 10.1111/j.1365-2664.2011.02048.x

[B5] Cantón Y. Del Barrio G. Solé-Benet A. Lázaro R. (2004). Topographic controls on the spatial distribution of ground cover in the tabemas badlands of se Spain. Catena 55, 341–365. doi: 10.1016/s0341-8162(03)00108-5

[B6] Cavender-Bares J. Kozak K. H. Fine P. V. A. Kembel S. W. (2009). The merging of community ecology and phylogenetic biology. Ecol. Lett. 12, 693–715. doi: 10.1111/j.1461-0248.2009.01314.x, PMID: 19473217

[B7] Chen Z. Huang Y. B. Zhu Z. P. Zheng Q. Q. Que C. X. Dong J. W. (2018). Landscape pattern evolution along terrain gradient in fuzhou city, fujian province, China. Ying Yong Sheng Tai Xue Bao 29, 4135–4144. doi: 10.13287/j.1001-9332.201812.010, PMID: 30584742

[B8] Chen L. Swenson N. G. Ji N. N. Mi X. C. Ren H. B. Guo L. D.. (2019). Differential soil fungus accumulation and density dependence of trees in a subtropical forest. Science 366, 124–128. doi: 10.1126/science.aau1361, PMID: 31604314

[B9] Chen S. R. Yang Z. Y. Wu Y. H. Li Y. Meng L. C. Chen L. Y.. (2024). Species prefer to shifting niche positions rather than expanding niche breadth to adapt to the heterogeneous karst forests. For. Ecosyst. 11, 100247. doi: 10.1016/j.fecs.2024.100247

[B10] Chiu M. C. Ao S. C. He F. Z. Resh V. H. Cai Q. H. (2020). Elevation shapes biodiversity patterns through metacommunity-structuring processes. Sci. Total Environ. 743, 140548. doi: 10.1016/j.scitotenv.2020.140548, PMID: 32758813

[B11] Cirimwami L. Doumenge C. Kahindo J.-M. Amani C. (2019). The effect of elevation on species richness in tropical forests depends on the considered lifeform: Results from an east african mountain forest. Trop. Ecol. 60, 473–484. doi: 10.1007/s42965-019-00050-z

[B12] Cornwell W. K. Ackerly D. D. (2009). Community assembly and shifts in plant trait distributions across an environmental gradient in coastal california. Ecol. Monogr. 79, 109–126. doi: 10.1890/07-1134.1

[B13] Deák B. Kovács B. Rádai Z. Apostolova I. Kelemen A. Kiss R.. (2021). Linking environmental heterogeneity and plant diversity: The ecological role of small natural features in homogeneous landscapes. Sci. Total Environ. 763, 144199. doi: 10.1016/j.scitotenv.2020.144199, PMID: 33383506

[B14] Delpiano C. A. Prieto I. Loayza A. P. Carvajal D. E. Squeo F. A. (2020). Different responses of leaf and root traits to changes in soil nutrient availability do not converge into a community-level plant economics spectrum. Plant Soil 450, 463–478. doi: 10.1007/s11104-020-04515-2

[B15] Dent D. H. Estrada-Villegas S. (2021). Uniting niche differentiation and dispersal limitation predicts tropical forest succession. Trends Ecol. Evol. 36, 700–708. doi: 10.1016/j.tree.2021.04.001, PMID: 33966918

[B16] Díaz S. Lavorel S. De Bello F. Quétier F. Grigulis K. Robson T. M. (2007). Incorporating plant functional diversity effects in ecosystem service assessments. Proc. Natl. Acad. Sci. U.S.A. 104, 20684–20689. doi: 10.1073/pnas.0704716104, PMID: 18093933 PMC2410063

[B17] Dornbush M. E. Wilsey B. J. (2010). Experimental manipulation of soil depth alters species richness and co-occurrence in restored tallgrass prairie. J. Ecol. 98, 117–125. doi: 10.1111/j.1365-2745.2009.01605.x

[B18] Dray S. Bauman D. Blanchet G. Borcard D. Clappe S. Guenard G.. (2020). Adespatial: Multivariate multiscale spatial analysis. Ecol. Monogr. 82. doi: 10.1890/11-1183.1

[B19] Du H. Hu F. Zeng F. P. Wang K. L. Peng W. X. Zhang H.. (2017). Spatial distribution of tree species in evergreen-deciduous broadleaf karst forests in southwest China. Sci. Rep. 7. doi: 10.1038/s41598-017-15789-5, PMID: 29142282 PMC5688135

[B20] Du H. Wang K. L. Peng W. X. Zeng F. P. Song T. Q. Zhang H.. (2014). Spatial heterogeneity of soil mineral oxide components in depression between karst hills, southwest China. Chin. Geographical Sci. 24, 163–179. doi: 10.1007/s11769-013-0630-9

[B21] Fu R. Y. Dai L. C. Zhang Z. H. Hu G. (2023). Community assembly along a successional chronosequence in the northern tropical karst mountains, south China. Plant Soil 491, 317–331. doi: 10.1007/s11104-023-06118-z

[B22] Gaston K. J. (2000). Global patterns in biodiversity. Nature 405, 220–227. doi: 10.1038/35012228, PMID: 10821282

[B23] Guilbeault-Mayers X. Lambers H. Laliberté E. (2024). Coordination among leaf and fine-root traits along a strong natural soil fertility gradient. Plant Soil. doi: 10.1007/s11104-024-06740-5

[B24] Hamm M. Drossel B. (2017). Habitat heterogeneity hypothesis and edge effects in model metacommunities. J. Theor. Biol. 426, 40–48. doi: 10.1016/j.jtbi.2017.05.022, PMID: 28529154

[B25] Hara M. Hirata K. Fujihara M. Oono K. (1996). Vegetation structure in relation to micro-landform in an evergreen broad-leaved forest on amami ohshima Island, South-West Japan. Ecol. Res. 11, 325–337. doi: 10.1007/BF02347790

[B26] He J. S. Wang X. P. Flynn D. F. B. Wang L. Schmid B. Fang J. Y. (2009). Taxonomic, phylogenetic, and environmental trade-offs between leaf productivity and persistence. Ecology 90, 2779–2791. doi: 10.1890/08-1126.1, PMID: 19886487

[B27] Hillerislambers J. Adler P. B. Harpole W. S. Levine J. M. Mayfield M. M. (2012). Rethinking community assembly through the lens of coexistence theory. Annu. Rev. Ecology Evolution Systematics 43, 227–248. doi: 10.1146/annurev-ecolsys-110411-160411

[B28] Hjort J. Gordon J. E. Gray M. Hunter M. L. Jr. (2015). Why geodiversity matters in valuing nature’s stage. Conserv. Biol. 29, 630–639. doi: 10.1111/cobi.12510, PMID: 25923307

[B29] Hu G. Pang Q. L. Hu C. Xu C. H. Zhang Z. H. Zhong C. F. (2024). Beta diversity patterns and determinants among vertical layers of tropical seasonal rainforest in karst peak-cluster depressions. Forests 15, 365. doi: 10.3390/f15020365

[B30] Hu G. Zhang Z. Wu H. Li L. (2023). Factors influencing the distribution of woody plants in tropical karst hills, south China. PeerJ 11, e16331. doi: 10.7717/peerj.16331, PMID: 37908415 PMC10615033

[B31] Huang J. (2025). Floristic characteristics and regionalisation of karst woody plants in China. Nordic J. Bot. 6, e04622. doi: 10.1111/njb.04622

[B32] Huang Y. T. Yao L. Ai X. R. Lü S. J. Ding Y. (2015). Quantitative classification of the subtropical evergreen-deciduous broadleaved mixed forest and the deciduous and evergreen species composition structure across two national nature reserves in the southwest of hubei, China. Chin. J. Plant Ecol. 39, 990. doi: 10.17521/cjpe.2015.0096

[B33] Jin L. Shi H. Y. Li T. Zhao N. Xu Y. Xiao T. W.. (2023). A DNA barcode library for woody plants in tropical and subtropical China. Sci. Data 10, 819. doi: 10.1038/s41597-023-02742-7u, PMID: 37993453 PMC10665436

[B34] Jones M. M. Tuomisto H. Borcard D. Legendre P. Clark D. B. Olivas P. C. (2008). Explaining variation in tropical plant community composition: Influence of environmental and spatial data quality. Oecologia 155, 593–604. doi: 10.1007/s00442-007-0923-8, PMID: 18064493

[B35] Kamrani A. Jalili A. Naqinezhad A. Attar F. Maassoumi A. A. Shaw S. C. (2011). Relationships between environmental variables and vegetation across mountain wetland sites, n. Iran. Biologia 66, 76–87. doi: 10.2478/s11756-010-0127-2

[B36] Keddy P. A. (1992). Assembly and response rules: Two goals for predictive community ecology. J. Vegetation Sci. 3, 157–164. doi: 10.2307/3235676

[B37] Keppel G. Van Niel K. P. Wardell-Johnson G. W. Yates C. J. Byrne M. Mucina L.. (2012). Refugia: Identifying and understanding safe havens for biodiversity under climate change. Global Ecol. Biogeography 21, 393–404. doi: 10.1111/j.1466-8238.2011.00686.x

[B38] Kraft N. J. B. Valencia R. Ackerly D. D. (2008). Functional traits and niche-based tree community assembly in an amazonian forest. Science 322, 580–582. doi: 10.1126/science.1160662, PMID: 18948539

[B39] Lai J. S. Zou Y. Zhang J. L. Peres-Neto P. (2022). Generalizing hierarchical and variation partitioning in multiple regression and canonical analysis using the rdacca.Hp r package. Methods Ecol. Evol. 13, 782–788. doi: 10.1111/2041-210X.13800

[B40] Laliberté E. Legendre P. Shipley B. (2014). Fd: Measuring functional diversity from multiple traits, and other tools for functional ecology. R Package version 1, 0–12. 10.1890/08-2244.1 20380219

[B41] Legendre P. Mi X. Ren H. Ma K. Yu M. Sun I.-F.. (2009). Partitioning beta diversity in a subtropical broad-leaved forest of China. Ecology 90, 663–674. doi: 10.1890/07-1880.1, PMID: 19341137

[B42] Legras G. Loiseau N. Gaertner J. C. Poggiale J. C. Gaertner-Mazouni N. (2020). Assessing functional diversity: The influence of the number of the functional traits. Theor. Ecol. 13, 117–126. doi: 10.1007/s12080-019-00433-x

[B43] Li K. Zhang M. Y. Li Y. L. Xing X. Y. Fan S. X. Cao Y.. (2020). Karren habitat as the key in influencing plant distribution and species diversity in shilin geopark, southwest China. Sustainability 12, 5808. doi: 10.3390/su12145808

[B44] Lian Z. W. Du H. Gu J. K. Zeng F. P. Peng W. X. Yin L. C.. (2023). Spatial heterogeneity of soil available medium- and micro-elements in evergreen-deciduous broadleaved forest in karst. J. Appl. Ecol. 34, 955–961. doi: 10.13287/j.1001-9332.202304.008, PMID: 37078313

[B45] Liang C. Z. Zhu Z. Y. Wang W. Pei H. Zhang T. Wang Y. L. (2004). The diversity and spatial distribution of plant communities in the helan mountains. Chin. J. Plant Ecol. 28, 361–368. doi: 10.17521/cjpe.2004.0052

[B46] Liu Z. L. Bi L. Z. Song G. H. Wang Q. B. Liu Q. Jin G. Z. (2017). Influence of topography on leaf area index in a typical mixed broadleaved-korean pine forest in xiaoxing’an mountains, China. Chin. J. Appl. Ecol. 28, 2856–2862. doi: 10.13287/j.1001-9332.201709.024

[B47] Liu C. N. Huang Y. Wu F. Liu W. J. Ning Y. Q. Huang Z. R.. (2021). Plant adaptability in karst regions. J. Plant Res. 134, 889–906. doi: 10.1007/s10265-021-01330-3, PMID: 34258691

[B48] Long Q. Z. Du H. Su L. Zeng F. P. Lian Z. W. Peng W. X.. (2023). Patterns in leaf traits of woody species and their environmental determinants in a humid karstic forest in southwest China. Front. Ecol. Evol. 11. doi: 10.3389/fevo.2023.1230819

[B49] Long W. X. Schamp B. S. Zang R. G. Ding Y. Huang Y. F. Xiang Y. Z. (2015). Community assembly in a tropical cloud forest related to specific leaf area and maximum species height. J. Vegetation Sci. 26, 513–523. doi: 10.1111/jvs.12256

[B50] Loreau M. Naeem S. Inchausti P. Bengtsson J. Grime J. P. Hector A.. (2001). Biodiversity and ecosystem functioning: Current knowledge and future challenges. Science 294, 804–808. doi: 10.1126/science.1064088, PMID: 11679658

[B51] Lu M. Z. Du H. Song T. Q. Peng W. X. Liu K. P. Su L.. (2021a). Characteristics of sprouting in woody plants in evergreen-deciduous broadleaf karst forest in mulun national nature reserve. Acta Ecologica Sin. 41, 6182–6190. doi: 10.5846/stxb202004291045

[B52] Lu M. Z. Du H. Song T. Q. Peng W. X. Su L. Zhang H.. (2021b). Effects of density dependence in an evergreen-deciduous broadleaf karst forest in southwest China. For. Ecol. Manage. 490, 119142. doi: 10.1016/j.foreco.2021.119142

[B53] Lv T. Ding H. Wang N. J. Xie L. Chen S. F. Wang D.. (2024). The roles of environmental filtering and competitive exclusion in the plant community assembly at mt. Huangshan are forest-type-dependent. Global Ecol. Conserv. 51, e02906. doi: 10.1016/j.gecco.2024.e02906

[B54] Manish K. (2021). Species richness, phylogenetic diversity and phylogenetic structure patterns of exotic and native plants along an elevational gradient in the himalaya. Ecol. Processes 10, 64. doi: 10.1186/s13717-021-00335-z

[B55] Mcgill B. J. Enquist B. J. Weiher E. Westoby M. (2006). Rebuilding community ecology from functional traits. Trends Ecol. Evol. 21, 178–185. doi: 10.1016/j.tree.2006.02.002, PMID: 16701083

[B56] Mcintire E. J. B. Fajardo A. (2009). Beyond description: The active and effective way to infer processes from spatial patterns. Ecology 90, 46–56. doi: 10.1890/07-2096.1, PMID: 19294912

[B57] Naeem S. Wright J. P. (2003). Disentangling biodiversity effects on ecosystem functioning: Deriving solutions to a seemingly insurmountable problem. Ecol. Lett. 6, 567–579. doi: 10.1046/j.1461-0248.2003.00471.x

[B58] Niu K. C. Liu Y. N. Shen Z. H. He F. L. Fan J. Y. (2009). Community assembly: The relative importance of neutral theory and niche theory. Biodiversity Sci. 17, 579–593. doi: 10.3724/SP.J.1003.2009.09142

[B59] Oksanen J. Blanchet F. G. Kindt R. Legendre P. Minchin P. O’hara B.. (2015). Vegan: Community ecology package. R Package Version 2.2-1 2, 1–2.

[B60] Pahlich E. Gerlitz C. (1980). A rapid DNA isolation procedure for small quantities of fresh leaf tissue. Phytochemistry 19, 11–13. doi: 10.1016/0031-9422(80)85004-7

[B61] Pérez-Harguindeguy N. Diaz S. Garnier E. Lavorel S. Poorter H. Jaureguiberry P.. (2016). Corrigendum to: New handbook for standardised measurement of plant functional traits worldwide. Aust. J. Bot. 64, 715. doi: 10.1071/BT12225_CO

[B62] Pichon B. Gounand I. Donnet S. Kéfi S. (2024). The interplay of facilitation and competition drives the emergence of multistability in dryland plant communities. Ecology 105, e4369. doi: 10.1002/ecy.4369, PMID: 38955486

[B63] Qi Y. C. Dong Y. S. Jin Z. Peng Q. Xiao S. S. He Y. T. (2010). Spatial heterogeneity of soil nutrients and respiration in the desertified grasslands of inner Mongolia, China. Pedosphere 20, 655–665. doi: 10.1016/S1002-0160(10)60055-0

[B64] Qin C. Tang M. Zhang X. M. (2025). Factors influencing natural regeneration of fagus hayatae. PeerJ 13, e19761. doi: 10.7717/peerj.19761, PMID: 40718773 PMC12296566

[B65] Rao M. D. Feng G. Zhang J. L. Mi X. C. Chen J. H. (2013). Effects of environmental filtering and dispersal limitation on species and phylogenetic beta diversity in gutianshan national nature reserve. Chin. Sci. Bull. (Chinese Version) 58, 1204. doi: 10.1360/972012-1582

[B66] Shen G. C. Yu M. J. Hu X. S. Mi X. C. Ren H. B. Sun I. F.. (2009). Species-area relationships explained by the joint effects of dispersal limitation and habitat heterogeneity. Ecology 90, 3033–3041. doi: 10.1890/08-1646.1, PMID: 19967859

[B67] Shen Z. H. Zhang X. S. Jin Y. X. (2000). Gradient analysis of the influence of mountain topography on vegetation pattern. Acta Phytoecologica Sin. 24, 430–435.

[B68] Smith S. A. O’meara B. C. (2012). Treepl: Divergence time estimation using penalized likelihood for large phylogenies. Bioinformatics 28, 2689–2690. doi: 10.1093/bioinformatics/bts492, PMID: 22908216

[B69] Stamatakis A. (2006). Raxml-vi-hpc: Maximum likelihood-based phylogenetic analyses with thousands of taxa and mixed models. Bioinformatics 22, 2688–2690. doi: 10.1093/bioinformatics/btl446, PMID: 16928733

[B70] Stein A. Gerstner K. Kreft H. (2014). Environmental heterogeneity as a universal driver of species richness across taxa, biomes and spatial scales. Ecol. Lett. 17, 866–880. doi: 10.1111/ele.12277, PMID: 24751205

[B71] Su L. Du H. Zeng F. P. Peng W. X. Wang H. Wang K. L.. (2023a). Environmental and spatial contributions to tree community assembly across life stages and scales in evergreen-deciduous broadleaf karst forests, southwest China. J. Forestry Res. 34, 1323–1331. doi: 10.1007/s11676-022-01587-x

[B72] Su L. Du H. Zeng F. P. Wang H. Lu M. Z. Luo L. J.. (2023b). Habitat associations of woody plant species in evergreen–deciduous broadleaf karst forests in southwest China. Front. Ecol. Evol. 11. doi: 10.3389/fevo.2023.1148910

[B73] Tamme R. Hiiesalu I. Laanisto L. Szava-Kovats R. Pärtel M. (2010). Environmental heterogeneity, species diversity and co-existence at different spatial scales. J. Vegetation Sci. 21, 796–801. doi: 10.1111/j.1654-1103.2010.01185.x

[B74] Tan S. S. Ye Z. L. Yuan L. B. Zhou R. F. Hu G. Jin X. F.. (2013). Beta diversity of plant communities in baishanzu nature reserve. Acta Ecologica Sin. 33, 6944–6956. doi: 10.5846/stxb201207010920

[B75] Tilman D. Wedin D. Knops J. (1996). Productivity and sustainability influenced by biodiversity in grassland ecosystems. Nature 379, 718–720. doi: 10.1038/379718a0

[B76] Toriyama J. Hak M. Imaya A. Hirai K. Kiyono Y. (2015). Effects of forest type and environmental factors on the soil organic carbon pool and its density fractions in a seasonally dry tropical forest. For. Ecol. Manage. 335, 147–155. doi: 10.1016/j.foreco.2014.09.037

[B77] Townsend A. R. Asner G. P. Cleveland C. C. (2008). The biogeochemical heterogeneity of tropical forests. Trends Ecol. Evol. 23, 424–431. doi: 10.1016/j.tree.2008.04.009, PMID: 18582987

[B78] Viana J. L. Dalling J. W. (2022). Soil fertility and water availability effects on trait dispersion and phylogenetic relatedness of tropical terrestrial ferns. Oecologia 198, 733–748. doi: 10.1007/s00442-022-05131-w, PMID: 35179630

[B79] Wang H. Chen L. Song M. Song T. Q. Zeng F. P. Peng W. X.. (2017). Spatial heterogeneity of soil phosphorus and potassium in a mixed evergreen and deciduous broad-leaved forest in karst region of southwest China. Acta Ecologica Sin. 37, 8285–8293. doi: 10.5846/stxb201611182348

[B80] Wang X. G. Swenson N. G. Wiegand T. Wolf A. Howe R. Lin F.. (2013). Phylogenetic and functional diversity area relationships in two temperate forests. Ecography 36, 883–893. doi: 10.1111/j.1600-0587.2012.00011.x

[B81] Wang X. G. Wiegand T. Anderson-Teixeira K. J. Bourg N. A. Hao Z. Q. Howe R.. (2018). Ecological drivers of spatial community dissimilarity, species replacement and species nestedness across temperate forests. Global Ecol. Biogeography 27, 581–592. doi: 10.1111/geb.12719

[B82] Wang Y. Zhang L. M. Chen J. Feng L. Li F. Yu L. F. (2022). Functional diversity of plant communities in relationship to leaf and soil stoichiometry in karst areas of southwest China. Forests 13, 864. doi: 10.3390/f13060864

[B83] Webb C. O. Ackerly D. D. Mcpeek M. A. Donoghue M. J. (2002). Phylogenies and community ecology. Annu. Rev. Ecol. Systematics 33, 475–505. doi: 10.1146/annurev.ecolsys.33.010802.150448

[B84] Wu Y. Y. Wu Y. S. (2023). The diversification of adaptive strategies for karst-adaptable plants and the utilization of plant resources in karst ecosystems. Agronomy 13, 2135. doi: 10.3390/agronomy13082135

[B85] Yao Y. C. Li E. G. Chen H. Y. Zhang J. H. Huang Y. M. (2017). Biodiversity of natural vegetation and influencing factors in western inner Mongolia. Biodiversity Sci. 25, 1303–1312. doi: 10.17520/biods.2017140

[B86] Yin D. Y. Ye Q. Cadotte M. W. (2021). Habitat loss-biodiversity relationships are influenced by assembly processes and the spatial configuration of area loss. For. Ecol. Manage. 496. doi: 10.1016/j.foreco.2021.119452

[B87] Zhang Z. H. Hu G. Zhu J. D. Luo D. H. Ni J. (2010). Spatial patterns and interspecific associations of dominant tree species in two old-growth karst forests, sw China. Ecol. Res. 25, 1151–1160. doi: 10.1007/s11284-010-0740-0

[B88] Zhang C. Zeng F. P. Zeng Z. X. Du H. Su L. Zhang L. J.. (2022). Impact of selected environmental factors on variation in leaf and branch traits on endangered karst woody plants of southwest China. Forests 13, 1080. doi: 10.3390/f13071080

[B89] Zhang L. X. Zhang F. Shangguan T. L. (2000). Vegetation diversity of luya mountains. Biodiversity Sci. 8, 361. doi: 10.17520/biods.2000051

