# Supplementary material for: Environmental filtering and dispersal limitation jointly shape the taxonomic, functional and phylogenetic diversity in a subtropical karst forest of China
Source: Front Plant Sci. 2025 Aug 28;16:1655071. doi: 10.3389/fpls.2025.1655071 (PMC12424234; doi:10.3389/fpls.2025.1655071)
Supplement: Supplementary file 1 [file DataSheet1.docx]

**Table.S1** Contribution of soil variables to principal components. Factor loadings with absolute values >0.50 are considered significant and indicate strong contribution to the principal component.

|  | TPC1 | TPC2 | TPC3 | TPC4 |
| --- | --- | --- | --- | --- |
| ELE | 0.8309 | 0.2901 | 0.1659 | -0.0659 |
| SLO | 0.8995 | 0.1338 | -0.2045 | -0.0377 |
| ASP | 0.4025 | 0.1699 | 0.1385 | 0.8760 |
| CON | 0.4011 | 0.5749 | 0.5157 | -0.3350 |
| TWI | -0.4342 | -0.2609 | 0.8086 | 0.0866 |
| STK | -0.6148 | 0.6415 | -0.1004 | 0.0901 |
| ROC | 0.6498 | -0.5837 | 0.1120 | -0.0562 |

**Table.S2** Contribution of topographic variables to principal components. Factor loadings with absolute values >0.50 are considered significant and indicate strong contribution to the principal component.

|  | SPC1 | SPC2 | SPC3 | SPC4 |
| --- | --- | --- | --- | --- |
| pH | 0.0357 | -0.5711 | 0.4351 | 0.4591 |
| SOC | 0.7815 | -0.0693 | -0.0220 | -0.1276 |
| TN | 0.7296 | -0.1308 | -0.0430 | -0.2489 |
| TP | 0.3137 | 0.6544 | 0.3020 | 0.0674 |
| TK | 0.0722 | 0.4576 | 0.6704 | -0.3484 |
| AN | 0.7288 | 0.0253 | 0.0564 | 0.0204 |
| AP | 0.2034 | 0.6349 | -0.1237 | 0.6050 |
| AK | 0.4528 | 0.2214 | -0.5503 | -0.1020 |
| Ca | 0.6876 | -0.1159 | 0.0712 | 0.2672 |
| Mg | 0.5602 | -0.4223 | 0.1323 | -0.0119 |


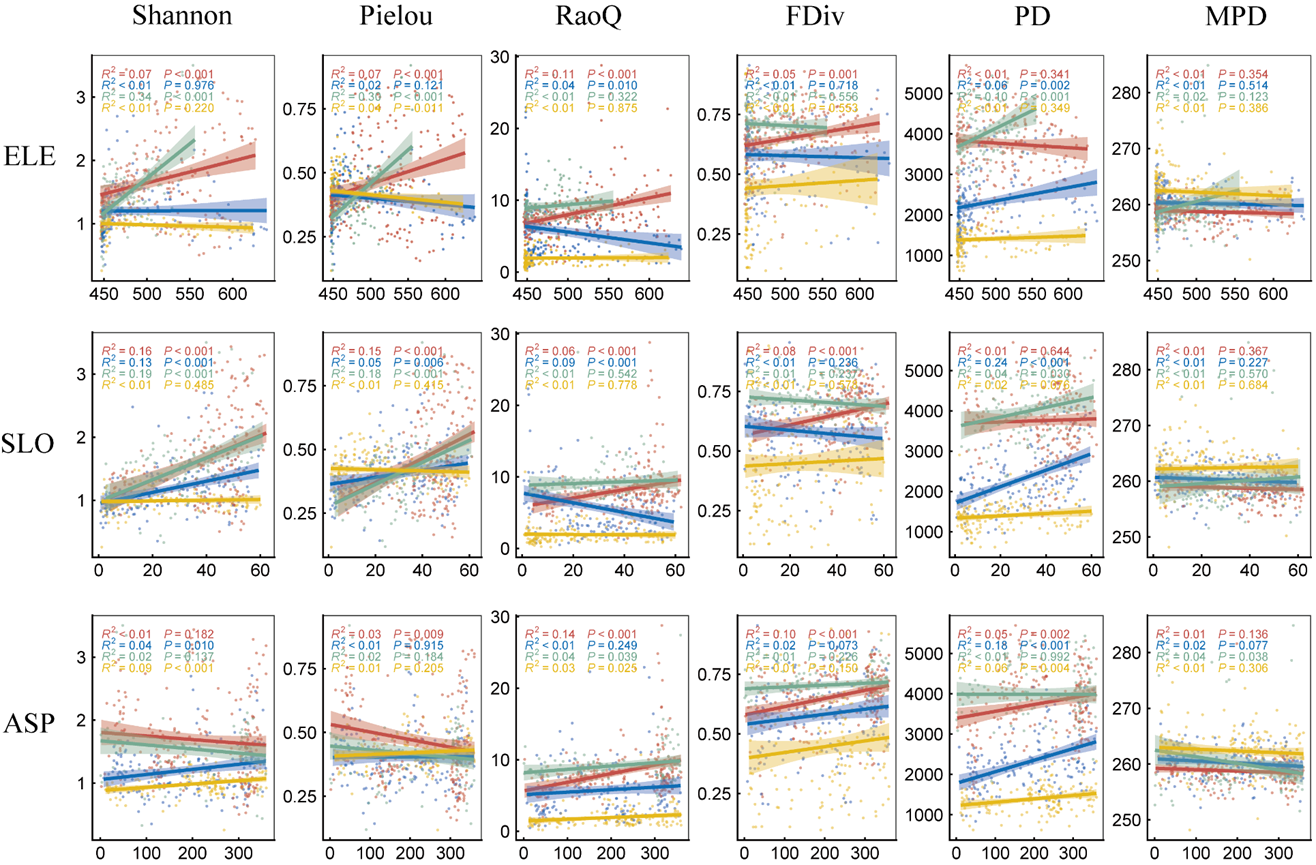


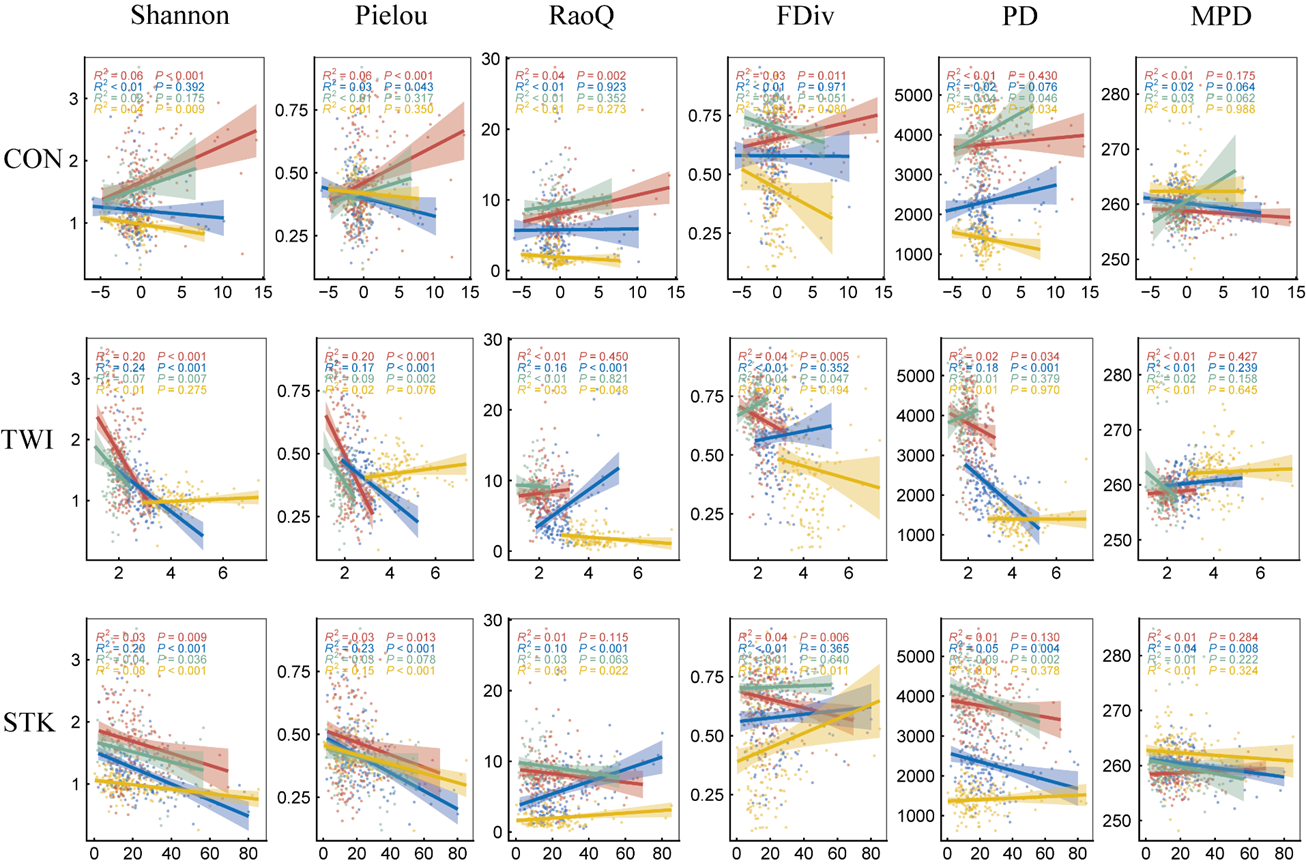

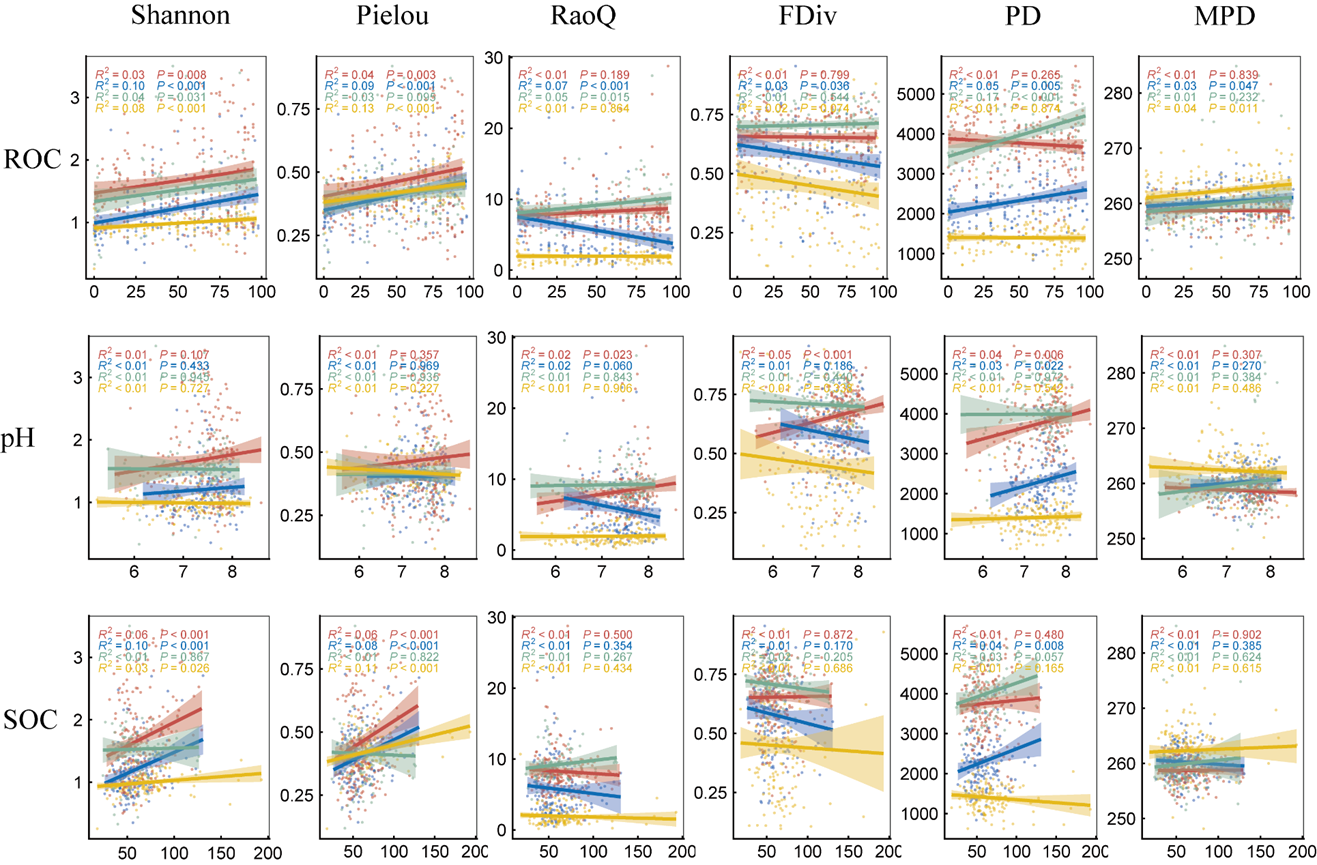

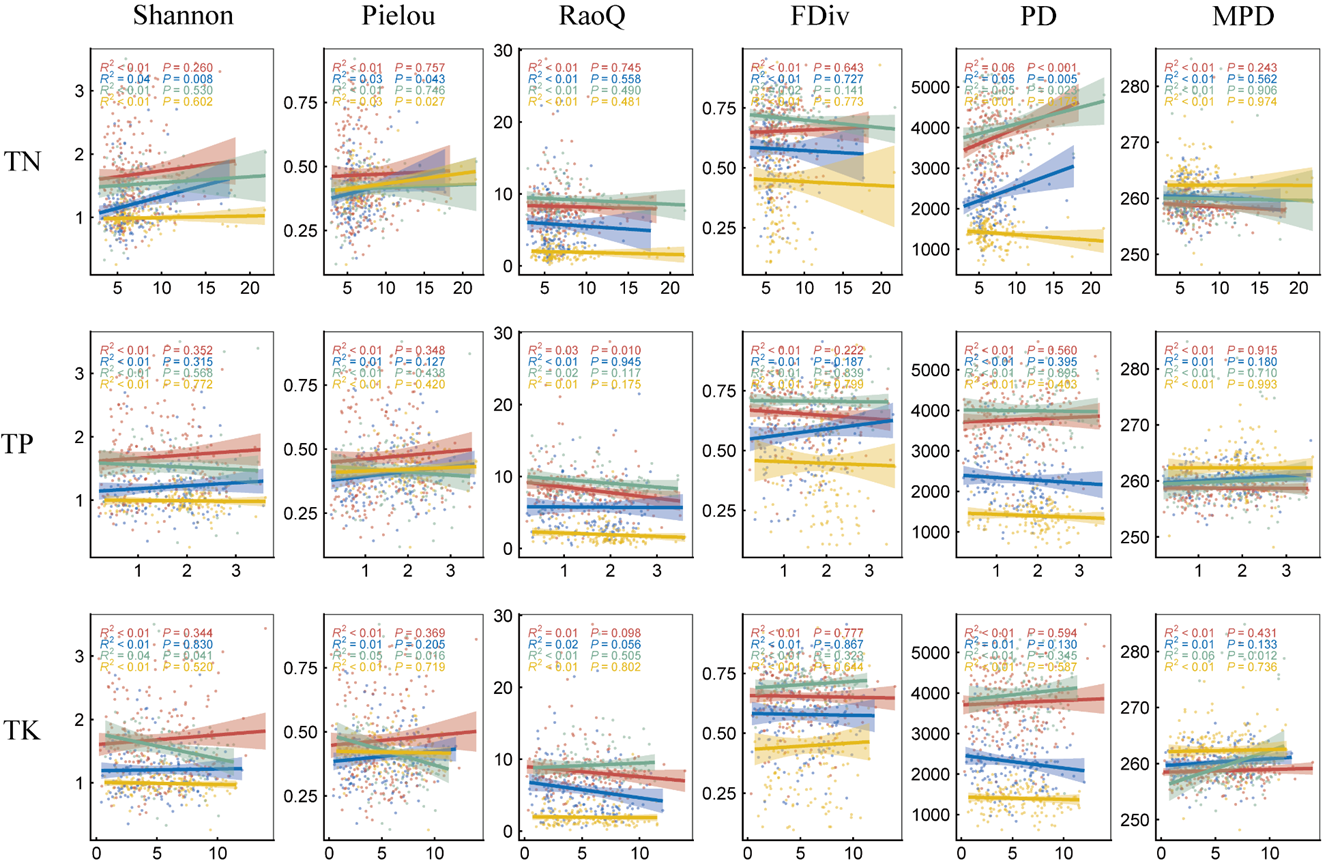

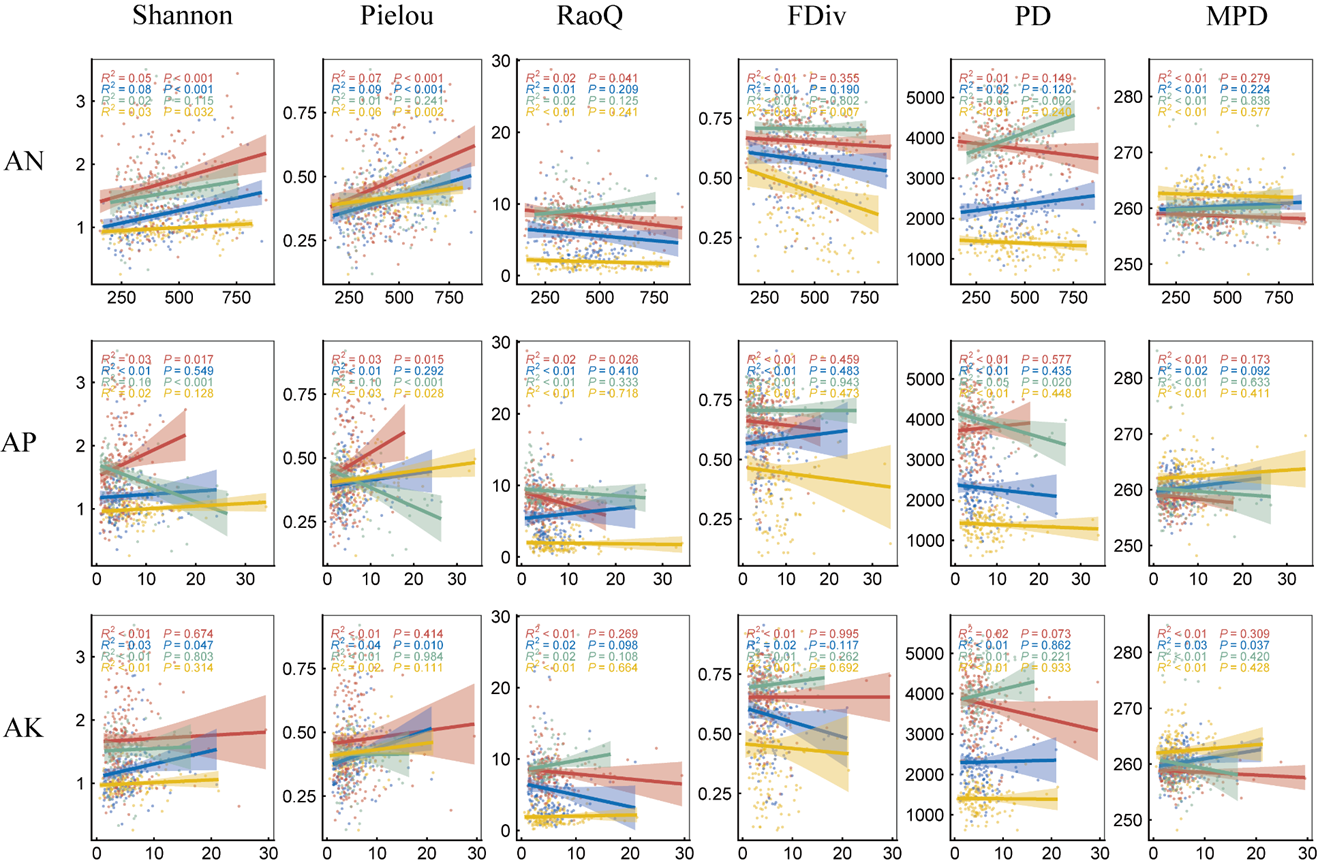

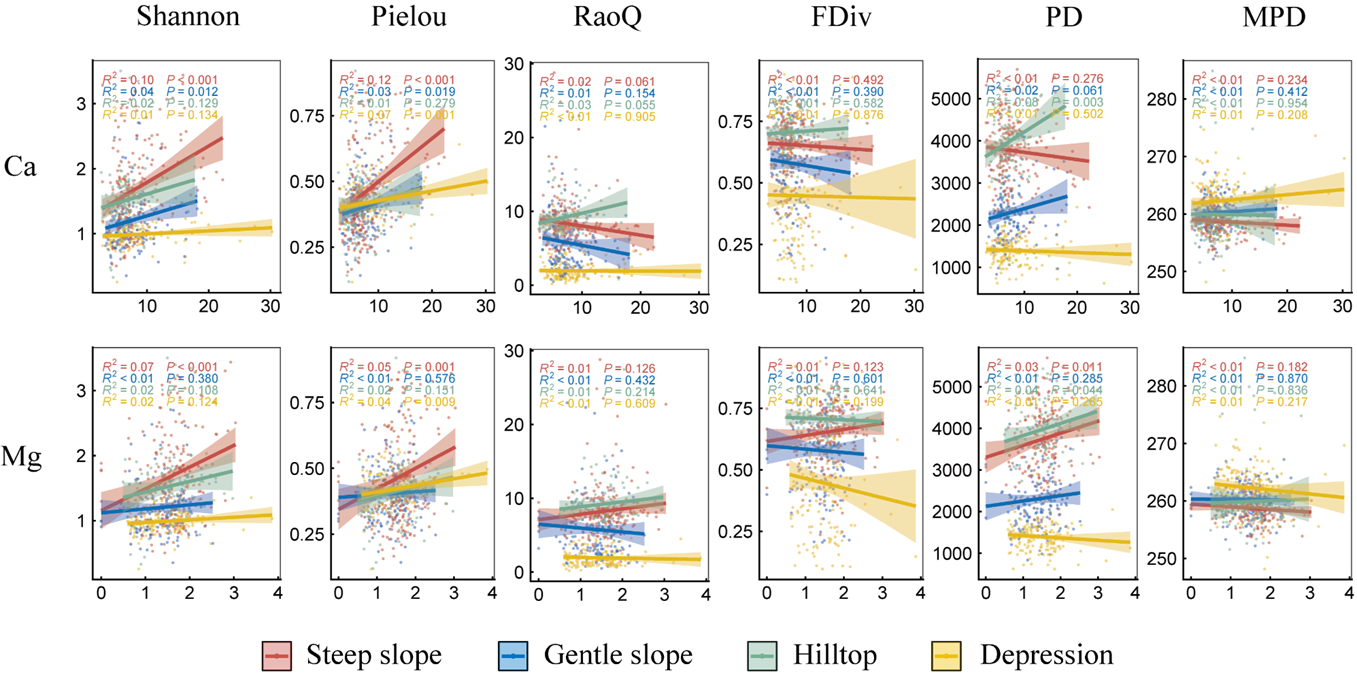


**Fig.S1** The relationship of taxonomic richness, phylogenetic and functional diversity with environmental factors. The lines show model predictions for significant parameter estimates based on the generalised linear mixed models and shading corresponds to 95% confidence intervals. Jittering was added for clarity, as well as transparency of points, darker areas thus indicate several accumulated points at the same or overlapping location.
